# Capabilities for Using Telemonitoring in Physiotherapy Treatment: Exploratory Qualitative Study

**DOI:** 10.2196/56432

**Published:** 2024-10-24

**Authors:** Charlotte van Westerhuis, Astrid F Sanders, Jesse J Aarden, Mel E Major, Marijke E de Leeuwerk, Nadine Florisson, Miriam H Wijbenga, Marike van der Schaaf, Marike van der Leeden, Maarten A van Egmond

**Affiliations:** 1 Physiotherapy Department, Faculty of Health, Sport and Physical Activity, Amsterdam University of Applied Sciences, Tafelbergweg 51, Amsterdam, 1105 BD, Netherlands, 31 634853608; 2 Ageing & Vitality, Amsterdam Movement Sciences, Amsterdam, Netherlands; 3 Physiotherapy Department, Hand and Wrist Center Amsterdam, Amsterdam, Netherlands; 4 Rehabilitation Medicine Department, Amsterdam UMC location Vrije Universiteit Amsterdam, Amsterdam, Netherlands; 5 Faculty of Science, Department of Health Sciences and Amsterdam Public Health Research Institute, Vrije Universiteit Amsterdam, Amsterdam, Netherlands; 6 Rehabilitation Medicine Department, Amsterdam UMC location University of Amsterdam, Amsterdam, Netherlands; 7 Musculoskeletal Health, Amsterdam Movement Sciences, Amsterdam, Netherlands

**Keywords:** telemedicine, telemonitoring, technology, physical therapy modalities, education, physiotherapist, physiotherapy, telehealth

## Abstract

**Background:**

Telemonitoring (TM), as part of telehealth, allows physiotherapists to monitor and coach their patients using remotely collected data. The use of TM requires a different approach compared with face-to-face treatment. Although a telehealth capability framework exists for health care professionals, it remains unclear what specific capabilities are required to use TM during physiotherapy treatments.

**Objective:**

This study aims to identify the capabilities required to use TM in physiotherapy treatment.

**Methods:**

An exploratory qualitative study was conducted following a constructivist semistructured grounded theory approach. Three heterogeneous focus groups were conducted with 15 lecturers of the School of Physiotherapy (Bachelor of Science Physiotherapy program) from the Amsterdam University of Applied Sciences. Focus group discussions were audiotaped and transcribed verbatim. Capabilities for using TM in physiotherapy treatment were identified during an iterative process of data collection and analysis, based on an existing framework with 4 different domains. Team discussions supported further conceptualization of the findings.

**Results:**

Sixteen capabilities for the use of TM in physiotherapy treatment were found addressing 3 different domains. Four capabilities were identified in the “digital health technologies, systems, and policies” domain, 7 capabilities in the “clinical practice and application” domain, and 5 capabilities in the “data analysis and knowledge creation” domain. No capabilities were identified in the “system and technology implementation” domain.

**Conclusions:**

The use of TM in physiotherapy treatment requires specific skills from physiotherapists. To best use TM in physiotherapy treatment, it is important to integrate these capabilities into the education of current and future physiotherapists.

## Introduction

In recent decades, the use of telehealth in health care has developed rapidly, both in terms of research and application [1,2]. The application of telehealth in health care varies widely from the use of wearable devices and health sensors to telecommunication and robotics [3]. Telemonitoring (TM), as part of telehealth, is the remote collection of information about patients’ health which is used to inform health care providers [4]. Since the COVID-19 pandemic, the use of TM by Dutch general practitioners has increased from 5% in 2019 to 30% in 2022 and will likely continue to increase to guarantee easy access to health care [5-7].

In physical therapy treatments, TM can support remote assessment, monitoring, and coaching of patients, such as through activity trackers to measure patients’ physical activity levels [4,8-10]. Data from activity trackers can provide insight into patients’ exercise behavior and treatment progress. For patients, this can increase their independence and self-management [11-16]. For physical therapists, it can support treatment decisions, such as by noticing changes early [12]. To tailor treatment specifically to the individual, it may be important to combine TM with traditional face-to-face treatment.

In general, combining traditional face-to-face treatment with telehealth-delivered care requires a different professional approach, necessitating advanced communication skills to compensate for the lack of visual cues and hands-on contact [17]. In physiotherapy, specific telehealth competencies need to be identified, clear guidance for specific technologies should be established, and a higher-order skill level is needed to combine traditional face-to-face treatment with telehealth [18].

Studies have outlined frameworks for the capabilities needed to deliver telehealth strategies in health care [17,19]. Brunner et al [20] identified capability statements for graduates and health professionals across four domains: (1) digital health technologies, systems, and policies; (2) clinical practice and applications; (3) data analysis and knowledge creation; and (4) system and technology implementation (Table 1). However, using telehealth within physiotherapy practice requires a more specific and professionally tailored approach [18]. Therefore Davies et al [17,21], specified capabilities for telehealth in physiotherapy with video consulting and telephone-based care focusing on compliance, patient privacy and confidentiality, patient safety, telehealth delivery, assessment and diagnosis, and planning and management. Although there are similarities between those interventions and TM, the use of TM goes beyond them since it allows the collection of continuous data of patients in their own environment. Making this valuable for treatment requires more knowledge and skills of TM technology, systems, and data, as well as a different coaching strategy [22]. The specific opportunities for using TM in physical therapy practice are not yet clear.

**Table 1.** Telehealth capability framework (Brunner et al [20]).

Domain	Capability statement
Digital technologies, systems, and policies	Understand the purpose and function of digital health technologies and systems implemented at a local, state, or national level, including consideration of legal, policy, and ethical implications.
Clinical practice and applications	Integrate digital health into clinical practice to deliver safe and quality care, including the provision of best practice models of care.
Data analysis and knowledge creation	Use data and data analysis to inform, deliver, and improve health and health care practice at an individual, team, or systems level.
System and technology implementation	Participate in digital health implementation, evaluation, and codesign processes to drive improvement and stimulate change.

Since capabilities are defined as “a holistic concept that describes how an individual or organization applies their ability in a confident manner to problems in new and unfamiliar circumstances as well as in familiar situations,” they may be more suitable as competency standards to frame eHealth skills in education [23]. With this, the concept of capabilities encompasses competence and goes beyond technical skills to emphasize adaptability to change, lifelong learning, and self-efficacy [24]. Further definition of TM capabilities for physiotherapists would help prepare current and future physiotherapists to use and implement TM, as well as provide guidance for physiotherapy education programs [20,22]. Therefore, this qualitative, exploratory study aims to define the required capabilities for using TM in physiotherapy treatment.

## Methods

### Design

For this qualitative, exploratory study among physiotherapy lecturers, we followed a constructivist semistructured approach using focus groups to collect data [25]. Group discussions focused on lecturers’ perceptions of the capabilities (future) physiotherapists required to use for TM. The COREQ (Consolidated Criteria for Reporting Qualitative Research) guidelines were used to ensure adequate reporting of the study [26].

### Participants and Sampling

We recruited lecturers from both the Dutch and international full-time Bachelor of Science Physiotherapy program (240 European Credit Transfer and Accumulation System [ECTS]) at the Amsterdam University of Applied Sciences (AUAS). A purposive sampling approach was applied to assemble a heterogeneous participant sample regarding diversity in age, work experience, and experience in TM, to enhance the transferability of the findings. Thirty-eight lecturers were invited by email to participate in the focus groups. Next, we applied snowball sampling by asking participants for potential eligible colleagues. Lecturers were eligible for inclusion if they (1) were working as physiotherapy lecturers at AUAS; (2) spoke Dutch to reflect on the telehealth education in the curriculum; and (3) were able to participate in the on-campus focus groups. Participation was completely voluntary.

### Data Collection

Between June and December 2022, 3 focus groups were organized, including 4‐6 participants each [25]. Group discussions took 90 minutes on average and were moderated by AS. NF supported AS, managed both tape recorders and time, and took notes. Additionally, an observer (CvW or MEdL) made sure every participant was given the opportunity to speak openly. If participants were not contributing, the observer invited those participants to share their thoughts or experiences. All researchers were familiar with the use of TM in health care.

The aim of the focus group was explained in the participant letter, as was the definition of TM; “Telemonitoring involves collecting data from the patient remotely and presenting it in an application or dashboard as part of their treatment. The data is collected without the physiotherapist physically seeing the patient.” Each participant was asked to individually answer 2 questions: “On a scale of 1‐10, to what extent are you supporting E-health applications in physiotherapy?“ and “How do you think TM is currently used in practice?,” before engaging in group discussion.

Focus groups followed a semistructured interview guide (Multimedia Appendix 1), using open-ended questions directed towards the topics of interest. Initial themes and topics were drawn from prior studies [27]. These were: (1) general experience with TM in clinical practice and education; (2) interpretation of TM data; (3) treatment time organization; (4) remote coaching; (5) being in charge versus autonomy; and (6) clinical reasoning with TM data. The interview guide was further developed through an iterative process, expanding topics about data interpretation to ensure data saturation, as these aspects were not specifically identified during the initial 2 focus groups.

### Data Analysis

Participant characteristics were described using quantitative data. All focus groups were audio recorded and transcribed verbatim by AS. AS and CvW performed qualitative data analysis and interpretation of the focus groups using a thematic grounded theory methodology, as described by the Qualitative Analysis Guide of Leuven, supported by MAXQDA 2022 [28,29]. AS and CvW performed open, axial, and selective coding, using a deductive approach [25]. After selective coding, initial capabilities were categorized into 4 higher-level domains, using the framework of Brunner et al [20], which describes capability statements across the domains of (1) digital health technologies, systems, and policies; (2) clinical practice and applications; (3) data analysis and knowledge creation; and (4) system and technology implementation (Table 1).

### Reflexivity

Credibility of data was achieved by data triangulation, using a questionnaire about lecturers’ experiences and thoughts of TM and member checking of the transcripts and narrative summary. The first and second focus groups were followed by a reflection meeting, to review the interview guide and moderator skills. One transcript was also read and coded by an independent researcher (MEM) to reflect on the core process of the analyzing researchers (AS and CvW). Transcripts were shared with participants for validation, before continuing data analysis [30]. We applied constant comparison between 2 researchers (AS and CvW) to ensure all information was included. Each coding phase included a team reflection meeting on the process and content of data analysis. An external educational researcher (MW) joined the research team after the initial analysis to help align initial results with educational principles and professional development standards. Additionally, several discussion team meetings helped formulate the specific capabilities. All changes and decisions during the research process were recorded in an audit trail, including field notes and memos.

### Ethical Considerations

The certified Ethics Research Committee of the AUAS approved the study protocol (ref #2022‐016637). Participants were informed about the aim of the study, and they were assured that their data would remain confidential and could not be traced back to them individually. They were informed that participation was voluntary and that they could stop at any time. All participants gave written informed consent before data collection started. To protect the privacy of the participants, all study-related data were deidentified and assigned a unique research identifier.

## Results

### Participants

We purposively invited a sample of 38 lecturers, of which 18 lecturers did not have time to join one of the focus groups and 3 lecturers did not respond to the invitation. A total of 17 lecturers were invited for a focus group, of which 15 lecturers participated in our focus groups, 1 lecturer was sick, and 1 lecturer did not show up. Details of the participants’ characteristics are provided in Table 2.

**Table 2.** Characteristics of participants (n=15).

Focus group number (duration) and participant number		Sex	Age (years)	Experience in TM^a^	Work experience as a lecturer (years)	Combines work as a lecturer with work as a physiotherapist in practice	Extent of support for telehealth use in physiotherapy practice^b^
**1 (95 min)**							
	P1	Female	34	Never used, never read about it	4	Yes	7
	P2	Male	42	Read about it	12	No	8
	P3	Female	47	Read about it	15	No	8
	P4	Male	54	Read about it	15	No	4
**2 (75 min)**							
	P5	Male	62	Using TM	21	Yes	10
	P6	Male	61	Using TM	15	Yes	9
	P7	Female	54	Never used, never read about it	1	No	8
	P8	Female	51	Never used, never read about it	2	No	8
	P9	Male	39	Using TM	13	Yes	8
**3 (75 min)**							
	P10	Female	34	Never used, never read about it	1	Yes	10
	P11	Male	53	Using TM	15	No	10
	P12	Female	39	Using TM and participating in research	1	Yes	7
	P13	Male	30	Read about it	1	Yes	7
	P14	Female	30	Using TM	2	Yes	8
	P15	Male	36	Read about it	1	Yes	8

a TM: telemonitoring.

b Support for telehealth in physiotherapy practice was rated on a scale from 0 to 10, where 0 indicates no support and 10 indicates full support.

### Capabilities

#### Overview

In the first step of open coding, a total of 316 codes were retrieved from all 3 focus groups. We identified 16 capabilities, that fitted into the first 3 domains of Brunner’s framework (Table 3), whereas the fourth domain did not reveal any match [20]. The categorization of these capabilities into the 3 domains is explained in more detail below, supported by quotes from participants (Table 3).

**Table 3.** Capabilities categorized according to the framework of Brunner et al [20] and quotations of interviewed participants.

Domain and capability		Quote number	Quotation	Participant number
**Digital health technologies, systems, and policies**				
	The physiotherapist is able to respond to different digital health-skills of patients	Q1	*You need to use different strategies with someone who has little understanding of their health and/or low literacy compared to someone who is highly literate with a high understanding, and all the mixes and combinations in between*.	5
	The physiotherapist is able to collect representative TM^a^ data	Q2	…*and also knowledge of how long you need to measure for before there is a notable difference to report. Imagine; you see a patient twice a week and you measure the number of steps for three days. That won’t say much about a particular data trend. You need to be aware of ‘what are my evaluation moments then?*	14
	The physiotherapist is able to critically consider the choice of digital metrics	Q3	*What is the goal? What do we want to achieve? We then have a sort of toolbox and I think TM is one of those tools alongside all kinds of other things*.	9
	The physiotherapist takes data protection regulations into account to guarantee privacy and safety of patient data	Q4	*You need to establish a clear contract agreeing on what happens with the data, who looks at it and who doesn’t, whether that’s within the practice or outside the practice, where it is stored, which computers are allowed access etc There’s really a whole different framework involved in documenting that.*	12
**Clinical practice and application**				
	The physiotherapist takes into account individual patient’s need for TM	Q5	*What does the patient really think? The treatment should be patient-centred, which is not (fully) what it is being done now.*	7
	The physiotherapist is able to instruct patients to understand and use TM measurements precisely	Q6	*Particularly in terms of exercise behavior, this is a tool that can be used with the goal of increasing the patient’s control and self-management. You have to observe whether the patient can manage by themselves, or if they need some assistance. It’s really a sort of coaching tool.*	8
	The physiotherapist is able to have a patient conversation about the collected TM data	Q7	*I have had experiences with patients where I received a set of data and said, “This can’t be right. I know that patient, I know him well. That’s impossible. This is so good that you could run a half marathon,” so something’s not right*.	11
	The physiotherapist finds the right balance between using the collected TM data and information provided by patients to formulate treatment strategies	Q8	*You may have more insight into the movement data of someone, but less into the person behind that data. I believe that the sweet spot is to find that balance, where you aim to combine the best of both sources*.	12
	The physiotherapist provides patients with insight regarding their personal TM data	Q9	*Many of our decisions are now based on the data which the patient also has access to. This way you can help the patient understand the exercise physiology in an engaging manner, so that after a couple times the patient can determine the next decision point on their own*.	6
	The physiotherapist combines current treatment methods with TM	Q10	*For me as a lecturer, telemonitoring could be used as an addition to what isn’t being done in the clinic, such as the patient receiving extra information. We can then incorporate this into the treatment process and revisit it when needed.*	3
	The physiotherapist is able to remotely encourage patients to achieve their treatment goals and support behavioral changes	Q11	*You can speak very technically to the patient, but if you don’t convey it in the right way at the right time, the patient won’t understand it*.	9
**Data analysis and knowledge creation**				
	The physiotherapist holds adequate experience and knowledge in digital measurement interpretation to establish correlations with current physiotherapy tests	Q12	*You do need to have basic statistical or mathematical understanding to recognize measurement errors and identify variables*.	11
	The physiotherapist is able to build advanced knowledge in exercise physiology, pathology, statistics, and epidemiology to accurately interpret data, surpassing the expertise of general physiotherapists	Q13	*You have to be statistically or mathematically literate to recognise the measurement errors. (...) That also requires a lot of education. Then there is also pathology-specific information*.	11
	The physiotherapist distinguishes between values within the norm and values outside the norm of collected TM data	Q14	*You have to know something about the clinical metrics (...). You will need to know what the norm is, what is deviant and also the reliability of the clinical metrics*.	1
	The physiotherapist is able to interpret data from the individual patient’s context	Q15	*A patient can train at 80% of their max test, and sometimes it’s doable, while other times it’s not. Because there are so many factors that influence heart rate – like emotions, temperature, medication, therapists need to have a good understanding and realize this data is not absolute*.	6
	The physiotherapist can reason from the level of TM data to patients’ body functions, activities, and participation level.	Q16	*I think students have difficulty making the link from body functions/disorders, to activities and participation level.(…) In terms of body functions/disorders, what are we actually measuring with TM data in terms of functions and what does it relate to directly?*	2
**System and technology implementation**		—^b^	—	—

a TM: telemonitoring.

b Not applicable.

#### Digital Health Technologies, Systems, and Policies

For patients to benefit from TM, physiotherapists should first objectively assess patients’ digital health skills at different levels and take appropriate actions (Q1). Additionally, physiotherapists need to understand the use, system, data collection, and interpretation procedures of TM to inform patients about its effectiveness and the need to collect representative TM data. To analyze data trends, it is essential to monitor patients over an extended time period. Therefore, it is crucial that patients understand the importance of data collection as they are responsible for their own data (Q2). Furthermore, critical consideration of the choice of digital metric is needed to underpin informed decision-making about the use of digital measurements in the patient’s context, in addition to regular treatment data (Q3). Given the sensitive nature of the collected data, it is imperative to consider data protection to guarantee the privacy and safety of patient data. Compliance with the General Data Protection Regulation law includes informing patients about who can access their data, where the data is stored, who can track patients with TM, and who is the official owner of the collected data (Q4).

#### Clinical Practice and Application

For TM data to support regular treatment, physiotherapists should understand the patients’ need for TM treatment*.* Blended care requires a distinct approach from traditional face-to-face treatment, due to the indirect nature of the interaction (Q5). For example, physiotherapists need to be able to instruct their patients to understand and use TM measurements accurately (Q6). Also, having a conversation with patients about discussing the collected TM data with the patient is a crucial step in effectively implementing TM into the treatment process. To optimize the benefits of TM data, it is important for physiotherapists to engage in a conversation with patients regarding the outcomes (Q7). This facilitates a holistic approach, where physiotherapists not only scrutinize the data itself but also consider the patient’s individual context. Lecturers agreed on the importance of striking a balance between the use of collected TM data and patient information to formulate treatment strategies (Q8). In addition to placing the data in the context of the patient, physiotherapists need to provide patients with insight into their personal TM data, to encourage patient autonomy in the use of TM data and personal treatment goals (Q9).

Blended care, including TM, requires physiotherapists to monitor patient data more frequently than with face-to-face appointments. Consequently, physiotherapists should change their current work routines, adopt a more patient-centered approach, and combine current treatment methods with TM (Q10). To enable a mix of face-to-face and remote patient coaching, physiotherapists will need to reorganize their work schedule to include multiple flexible contact moments for evaluation. In addition, they may need to learn more about behavior change techniques (BCTs) to remotely encourage patients to achieve their treatment goals (Q11).

#### Data Analysis and Knowledge Creation

To facilitate data analysis, physiotherapists need adequate experience and knowledge of digital measurement interpretation to establish correlations with current physiotherapy tests (Q12). Furthermore, physiotherapists should obtain advanced knowledge of exercise physiology, pathology, statistics, and epidemiology to accurately interpret data, beyond the expertise of general physiotherapists, to accurately interpret TM data (Q13). Due to the abundance of variables that can potentially affect the measurement, such as heart rate, emotions, temperature, medication, and illness, it seems that the basic knowledge of a graduated Bachelor of Science may not be sufficient to interpret TM data correctly. To ensure accurate interpretation, it is essential that physiotherapists can compare with norm values (Q14). Additionally, knowledge of the patient’s context is crucial, as is relating TM data to patients’ body functions, activities, and participation level for correct interpretation and follow-up of individual treatment goals (Q15, Q16). According to the participants, narrative and clinical reasoning are integral to this process. The physiotherapist needs to integrate TM data to help solve the health-seeking question, and support clinical decision-making. Clinical reasoning also underlies the information that is shared with the patient through the application with the final aim of accomplishing individualized treatment goals.

#### System and Technology Implementation

The focus group interviews did not provide adequate answers concerning the necessary capabilities to engage in digital health implementation, evaluation, and co-design.

## Discussion

### Principal Findings

This study identified 16 capabilities physiotherapists need to use TM in their daily treatment. These capabilities can help to identify and guide the development of necessary skills, thereby supporting the implementation of TM in physiotherapy treatment. The identified capabilities cover 3 distinct domains of Brunner’s framework [20].

Four capabilities were found to be linked with the domain of digital technologies, systems, and policies. These include the ability of a physiotherapist to objectively assess patients’ digital health literacy, which is crucial for optimal selection and tailoring of eHealth to the patients’ competencies [8,31,32]. Validated instruments are available within psychology, but they are not yet widely used in physiotherapy [2]. In a recent publication, Kloek et al [8] introduced the Dutch Blended Physiotherapy Checklist to guide physiotherapists in tailoring personalized blended treatments. Nevertheless, more research is needed to assess the feasibility and predictive validity of the checklist to inform further implementation in physiotherapy education and clinical practice.

The 7 capabilities identified within the domain of clinical practice and application relate to explaining, motivating, and coaching patients based on TM data. Here, the use of different BCTs by physiotherapists can be of value. Previous research has shown that integrating different BCTs with digital interventions improves physical activity, autonomy, and self-efficacy [10,22,33,34]. The combination with TM could therefore be a good alternative for real-life contact in practice, since it provides insight into continuous data, such as physical activity, heart rate, and well-being. Based on this, TM can facilitate goal setting and receive automatic feedback [34]. When communication about the data takes place remotely, there is some overlap with telephone-based care capabilities [17]. However, remote coaching using TM requires different skills from the physiotherapist compared with BCTs in one-to-one situations and only teleconsulting. This specifically involves informing the patient about TM, ensuring that they understand TM, making its goals clear, and conveying its added value [10,22,33,34]. Our study showed that it is not only necessary to initiate conversations about TM prior to its use, but also to continue these conversations throughout the treatment process. This ongoing dialogue is crucial to improve treatment adherence, self-management, and effectiveness.

Five capabilities were identified in the data analysis and knowledge creation domain. Recent studies showed that the lack of knowledge among health care providers is a barrier to the use of technology in health care. Different aspects of knowledge were distinguished: the technology system or technology application and the determination of optimal combined treatment for the individual patient [8,22,31]. A new aspect mentioned by our participants is the need for advanced knowledge of exercise physiology, pathology, statistics, and epidemiology to correctly interpret TM data and discover associations within and between the data of specific patients [35]. In addition, there is a need to improve knowledge about combining TM results with the patients’ body functions, activities, and participation levels. These topics can be included in training related to TM for (future) physiotherapists.

Even though no capability was formulated in the domain system and technology implementation, this does not mean that it is not important since barriers to the implementation of eHealth are well known [36,37]. How to address these barriers and what capabilities of physiotherapists are needed to do so may be a topic for further research.

An undiscovered theme in our focus groups is the role of TM as a facilitator for interprofessional collaboration. The use of TM potentially can promote interprofessional collaboration by collecting and combining data from different professionals, thus enhancing clinical reasoning and interdisciplinary goal setting, and ultimately improving health care delivery [22]. This aspect of TM was only marginally discussed in our focus groups. Still, it is essential to have sufficient expertise regarding the data collected, including TM data gathered by other health care professionals, to establish shared goals [13,38].

To comprehensively assess the capabilities of utilizing TM in physiotherapy, we formed focus groups comprising lecturers of varying ages and experience. Although we hypothesized that younger lecturers, would have better TM skills, more experienced participants demonstrated high proficiency in TM and advocated for its use. This aligns with findings by Lam et al [39] and Wentink et al [3], suggesting that younger physiotherapists may struggle with technology adoption in unfamiliar health care settings. Additionally, our participants emphasized the complex nature of TM data, which includes physiology, pathology, statistics, and epidemiology, which may pose challenges to students interning in clinical settings. Our lecturers mentioned that the ability to understand and incorporate TM data into clinical decision-making is acquired through practical experience. Therefore, the integration of these capabilities into physiotherapy education is essential.

### Strengths and Limitations

Although other studies had already identified the required capabilities for the use of telehealth, telephone-based care, and video-consulting in physiotherapy, our study provided specific insight into the use of TM in physiotherapy [17,18,20,21]. To our knowledge, this is the first study that identified relevant capabilities for physiotherapists to support the use and implementation of TM in physiotherapy treatment, based on existing theoretical and educational orientated frameworks [20]. The findings are relevant to future and current physiotherapists and may prompt educational physiotherapy programs to consider developing such skills as part of their curriculum. However, future research is needed to strengthen the educational approach. The focus groups were only conducted amongst lecturers at AUAS, therefore the results are limited to a single institution. To include multiple perspectives and develop a more comprehensive understanding, we recommend expanding the pool of participants to include lecturers from different institutions as well as working physiotherapists and students.

### Practical Relevance

Based on the capabilities identified in this study, we propose 2 new competencies for general physiotherapists within their role of health care provider, with a specific focus on the use of TM during physiotherapy treatment [40]. First, it is necessary for physiotherapists to adequately instruct their patients on how to collect continuous data, such as physical activity, heart rate, and well-being, while being able to recognize both normal and deviating measurements. An integrative approach to contextual factors and TM data is fundamental to this process since it allows physiotherapists and patients to correlate clinical and TM data and evaluate treatment outcomes. Second, physiotherapists must be attuned to patients’ needs and skills required to receive digital treatment. This will allow them to enhance their patients’ digital health skills and promote patients’ self-efficacy empowerment by informing them about the use and implementation of TM as part of individualized treatment.

### Conclusion

Our study suggests that the application of TM in physiotherapy treatment requires different capabilities compared with regular treatment. To optimally integrate TM into physiotherapy treatment, we recommend incorporating these findings into the practical education of current and future physiotherapists. This can be achieved by updating curricula in physiotherapy programs to include TM, and by offering specialized workshops and modules that focus on digital health technologies, clinical application, and data analysis. In addition, TM specialized courses can be organized to ensure that current physiotherapists will be educated in the use of TM. However, since our results are based on only lecturers of one University of Applied Sciences in the Netherlands, and 9 of the 15 lecturers had no experience with TM in practice, further research with lecturers with more clinical TM experiences and consumers is needed.

## Supplementary material

10.2196/56432 Multimedia Appendix 1 Interview guide.

## Abbreviations

AUAS: Amsterdam University of Applied Sciences
BCT: behavior change technique
COREQ: Consolidated Criteria for Reporting Qualitative Research
ECTS: European Credit Transfer and Accumulation System
TM: telemonitoring
