# Supplementary material for: Capabilities for Using Telemonitoring in Physiotherapy Treatment: Exploratory Qualitative Study
Source: JMIR Rehabil Assist Technol. 2024 Oct 24;11:e56432. doi: 10.2196/56432 (PMC11527389; doi:10.2196/56432)
Supplement: Multimedia Appendix 1 [file rehab-v11-e56432-s001.docx]

| **Main questions** | **Sub questions** |
| --- | --- |
| 1. How do you think TM can be used in physiotherapy clinical practice? |  |
| 1. What knowledge, skills, and attitude does a physiotherapist need to choose which data will be collected with TM? | What knowledge, skills, and attitude does a physiotherapist need to distinguish between main- and side topics? |
| 1. What knowledge, skills, and attitude does a physiotherapist need to know and understand the application? | What knowledge, skills, and attitude does a physiotherapist need to identify measurement errors?  How can a physiotherapist reflect on the quality of the collected data?  What knowledge, skills, and attitude does a physiotherapist need in order choose the right variable during TM?  Which knowledge about a TM application is required to use it? |
| 1. What knowledge, skills, and attitude does a physiotherapist need to interpret the data? | What knowledge and skills does a physiotherapist need to translate the data to the patient?  What knowledge does a physiotherapist need to use the data in clinical practice? |
| 1. What knowledge, skills, and attitude does a physiotherapist need to use data for shared decision making? | What knowledge, skills, and attitude does a physiotherapist need to understand the way data is collected for TM?  What knowledge, skills, and attitude does a physiotherapist need to use TM to collect data for future use (AI/big data)?  What does a physiotherapist need (skills and knowledge) to compare data between patients? |
| 1. What will change in privacy when you monitor your patient remotely? | What knowledge, skills, and attitude does a physiotherapist need to use a commercial product as a healthcare professional?  What knowledge, skills, and attitude does a physiotherapist need to use TM following the privacy rules?  What knowledge, skills, and attitude does a physiotherapist need to collect data for a data base / big data? |
| 1. How will you decide TM fits with the patient healthcare question and personal environment? | What knowledge, skills, and attitude does a physiotherapist need to make patient specific treatment goals with data from TM?  What knowledge, skills, and attitude does a physiotherapist need to change patients’ healthcare goals based on TM data?  How will you teach a student to reflect on treatment goals based on TM data? |
| 1. What is the most important thing you want to bring into education after this meeting? |  |
